# Erythema ab igne

**DOI:** 10.4269/ajtmh.14-0474

**Published:** 2015-03-04

**Authors:** Yulia Treister-Goltzman, Roni Peleg

**Affiliations:** The Department of Family Medicine and Siaal Research Center for Family Practice and Primary Care, Faculty of Health Sciences, Ben-Gurion University of the Negev, Israel; Beer-Sheva and Clalit Health Services, Southern District, Israel

**Figure 1.**
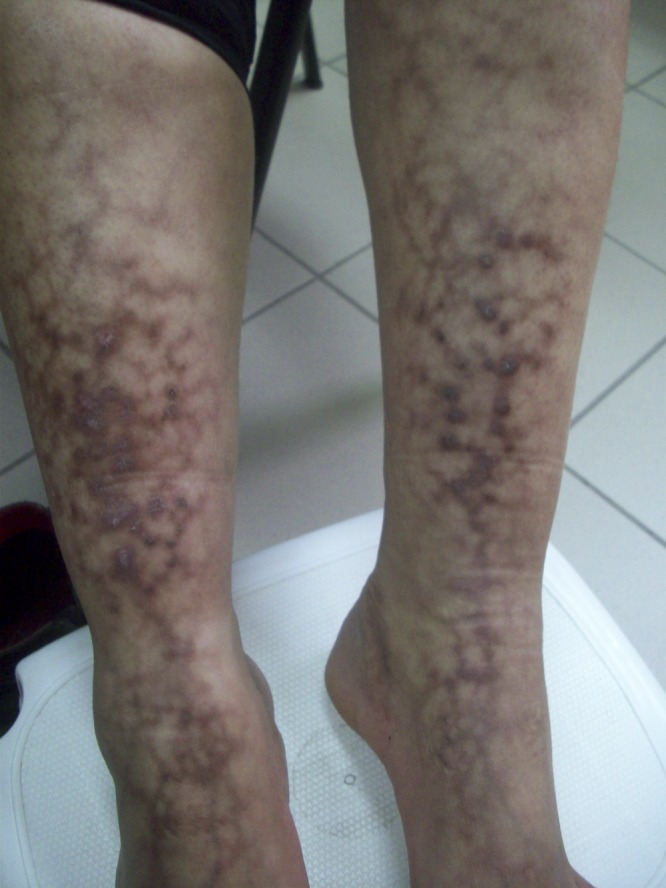
The rash on patient's shins and ankles.

A 30-year-old Bedouin woman who lives in a hut presented with skin changes in the anterior portion of both shins. The lesions were mildly itchy. The skin changes had developed gradually over some years and were significantly worse during winter periods. History revealed repeated sitting in front of an open fire in the hut used for warmth in winter. Physical examination (Figure 1) showed nonblanching brown lichenified hyperpigmentation in a reticular pattern on both anterior shins and ankles. Based on the findings a diagnosis of Erythema ab igne (EAI) was established.

Diagnosis of EAI is based on clinical history and physical examination.^1^ The EAI is caused by repeated exposure to heat at a lower level than that which causes a thermal burn (infrared radiation). Historically, this condition was seen in people, who sat closely to open fires. The EAI is a rare condition because of the advent of central heating. Its incidence has been rising as heating sources are being used to treat chronic pain. Currently, it is more commonly seen after repeated use of heating pads, laptops occupational exposure, and car heaters.^2^

Chronic lichenified lesions of EAI have potential for malignant transformation. A skin biopsy performed revealed no evidence of malignancy.

Erythema ab igne may be considered an infectious disease mimic. In an era in which health care workers are increasingly being globalized and deployed to exotic locations, lesions such as in the patient presented here may be misinterpreted because of lack of awareness of certain culture practices and conditions.
